# Core–Shell Eudragit S100 Nanofibers Prepared via Triaxial Electrospinning to Provide a Colon-Targeted Extended Drug Release

**DOI:** 10.3390/polym12092034

**Published:** 2020-09-07

**Authors:** Yanfei Ding, Cheng Dou, Shuyue Chang, Zhengming Xie, Deng-Guang Yu, Yanan Liu, Jun Shao

**Affiliations:** 1 School of Materials Science & Engineering, University of Shanghai for Science & Technology, 516 Jungong Road, Shanghai 200093, China; 182442519@st.usst.edu.cn (Y.D.); 182442525@st.usst.edu.cn (C.D.); 1826410301@st.usst.edu.cn (S.C.); 1826410106@st.usst.edu.cn (Z.X.); yananliu@usst.edu.cn (Y.L.); 2 Shanghai Institute of Technical Physics, Chinese Academy of Sciences, 500 Yutian Road, Shanghai 200083, China

**Keywords:** triaxial electrospinning, colon-targeted, extended-release, core–shell structure, aspirin

## Abstract

In this study, a new modified triaxial electrospinning is implemented to generate an Eudragit S100 (ES100)-based core–shell structural nanofiber (CSF), which is loaded with aspirin. The CSFs have a straight line morphology with a smooth surface, an estimated average diameter of 740 ± 110 nm, and a clear core–shell structure with a shell thickness of 65 nm, as disclosed by the scanning electron microscopy and transmission electron microscopy results. Compared to the monolithic composite nanofibers (MCFs) produced using traditional blended single-fluid electrospinning, aspirin presented in both of them amorously owing to their good compatibility. The CSFs showed considerable advantages over the MCFs in providing the desired drug-controlled-release profiles, although both of them released the drug in an erosion mechanism. The former furnished a longer time period of time-delayed-release and a smaller portion released during the first two-hour acid condition for protecting the stomach membranes, and also showed a longer time period of aspirin-extended-release for avoiding possible drug overdose. The present protocols provide a polymer-based process-nanostructure-performance relationship to optimize the reasonable delivery of aspirin.

## 1. Introduction

Electrospinning, as a peer technology of electrospraying, can create polymeric nanofibers with variable structural features in a single-step process [1,2,3,4,5,6,7], and should thus hold great promises for developing new types of polymer-based nanofibers loaded with different kinds of active ingredients [8,9,10,11], including those for treating coronavirus disease 2019 (COVID-19) [12,13,14]. In 1996, electrospinning (previously electrostatic spinning) and nanofibers were revived by Reneker’s group [15]. In 2002, the first publication of the applications of electrospun non-woven fiber mats for providing a drug-extended-release profile was reported [16]. From then on, electrospun medicated nanofibers for pharmaceutical applications have always been increasing until now [17,18,19,20,21]. A simple search in the Web of Science with “electrospinning or nanofibers and drug delivery” as the main topic can find 43,557 items (8 August 2020). Among these publications, almost all kinds of drug-controlled-release profiles can be provided by electrospun nanofibers and special nanostructures. These controlled-release profiles include immediate release [22], pulsatile release [23], sustained release [24], multiple-phase release [25], dual-stage release [26], extended release [27], delayed release [28], topical release [29], targeted release [30], and so on. These release profiles are mainly realized by the electrospinnable polymers’ physical and chemical properties [31], and also by the multiple-compartment structures such as core–shell, Janus, and their combinations [32,33]. 

In clinical applications, non-steroidal anti-inflammatory drugs (NSAIDs) are medicines widely exploited to alleviate fever, pain, and inflammation, which are the most common symptoms of COVID-19 patients [13,34,35]. Among all the NSAIDs, aspirin is one of the most frequently utilized candidates for treating the patients with COVID-19 infection through oral administration [14,36,37]. However, aspirin is a weak acid. When pH < 3.5, aspirin is deionized and becomes liposoluble [38]. Then, it is able to diffuse into mucosal cells and changes into an ionic form, causing mucosal cell damage [39]. Therefore, enteric coating of aspirin is a common way for the commercial products to reduce mucosal damage [40,41].

Here, we hypothesize a new kind of core–shell structural nanofiber (CSF), which can manipulate the release of aspirin through a colon-targeted way and an extended manner after oral administration. The commercial pH-sensitive copolymer Eudragit S100 (ES100), which has a pH-dependent solubility [42], was exploited as the polymeric carrier and also the filament-forming matrix. A modified triaxial electrospinning [43,44,45,46,47,48,49,50] was developed to create the core–shell ES100 nanofibers, in which a drug-free shell layer of ES100 is intentionally coated on a composite aspirin-ES100 core. The monolithic composite nanofibers (MCFs) with aspirin homogeneously distributed all over the ES100 matrix were prepared using a traditional blended electrospinning and were exploited as control.

## 2. Materials and Experiments 

### 2.1. Materials

Aspirin (purity of 99%) was obtained from Huashi Pharmacy (Shanghai, China). Eudragit^®^ S100 (Mw = 135,000) was bought from Röhm GmbH & Co. KG (Darmstadt, Germany). The color markers (including basic fuchsin and methylene blue) and the solvents (including anhydrous ethanol and *N*,*N*-dimethylacetamide, DMAc) were purchased from Shanghai Reagent Factory (Shanghai, China). Other chemicals were of analytical grade. Water was distilled doubly. 

### 2.2. Modified Triaxial Electrospinning

Three sorts of solutions were prepared using the same mixture (ethanol:DMAc = 8:2 % *v/v*). For the preparation of core–shell structural fibers (CSFs) through modified triaxial electrospinning, the outer fluid was the solvent mixture, the middle fluid consisted of 15 g ES100 in 100 mL solvent mixture (meaning an ES100 concentration of 15 % w/v), and the core fluid comprised 15 g ES100 and 5 g aspirin in 100 mL solvent mixture (meaning an ES100 concentration of 15 % w/v and an aspirin content of 5 % *w*/*v*). For optimization and observation of the experimental condition during the pre-experiments, 10⁻^4^ mg/mL methylene blue, 10⁻^3^ mg/mL basic fuchsin, and 10⁻^3^ mg/mL methylene blue were dissolved into the outer, middle, and core fluids, respectively. 

A homemade trifluid electrospinning apparatus was developed to carry out the modified tri-axial electrospinning for producing the CSFs, which comprised three fluid pumps (KDS100, Cole-Parmer, Vernon Hills, IL, USA), a high power supply (ZGF 60 kV/2 mA, Wuhan Hua-Tian Corp., Wuhan, China), a fiber collector composed of a cardboard box covered by an aluminum foil, and a detachable tri-layer concentric spinneret. A metal capillary with an inner diameter of 0.3 mm was exploited as a monoaxial spinneret for producing the MCFs. The spinneret-collector distance was fixed at 20 cm, and other parameters are listed in Table 1.

### 2.3. The Morphologies and Inner Structures of the MCFS and CSFs 

The morphologies of the MCFs and CSFs were assessed by scanning electron microscopy (SEM, Quanter 450, FEI, Fremont, CA, USA). Before the assessments, the nanofibers were stuck on a conductive adhesive, and then sputtered with a layer of gold for 60 min. The diameters of nanofibers can be estimated through the ImageJ software. The inner structural features of the MCFs and CSFs were determined through transmission electron microscopy (TEM, JEM 2100F, JEOL, Tokyo, Japan). 

### 2.4. Aspirin Physical State and its Compatibility with ES100 

The physical state of aspirin encapsulated in the MCFs and CSFs was measured using a Bruker X-ray diffractometer (Karlsruhu, Germany) with Cu Kα radiation. The range of 2θ in the achieved patterns was from 5 to 60°. The physical interactions between ES100 and aspirin were detected using a Spectrum 100 FTIR Spectrometer (PerkinElmer, Billerica, MA, USA) in the range of 4000 to 500 cm⁻^1^.

### 2.5. Functional Performances of the Colon-Targeted Extended Release of Aspirin

The Method II of Chinese Pharmacopoeia (2015 Ed.), i.e., the paddle method, was utilized to guide the in vitro dissolution tests of the aspirin released from the MCFs and CSFs. To mimic the pH situations in human digestive tracts, the dissolution media in the first two hours were HCl solution (artificial gastric fluid without enzyme, pH = 2.0, 0.01 M, 900 mL), which was later changed to 900 mL phosphate buffer solution (pH 7.4, 0.1 M, artificial colon fluid without enzyme). The dissolution media were kept at a fixed temperature of 37 ± 1 °C and the rotation rate of the paddle was 50 rpm.

The beginning time point was the time when the nanofibers were immersed into the dissolution media. A five milliliter aliquot was withdrawn at each predetermined time point, and 5.0 mL of the same fresh dissolution was compensated to maintain a constant volume of the dissolution media. The concentration of aspirin in the dissolution sample was determined using a UV-vis spectrophotometer (UV-2102PC, Unico Instrument Co. Ltd., Shanghai, China) at λ_max_ = 278 nm. All the measurements were repeated 6 times.

## 3. Results and Discussion

### 3.1. The Triaxial Electrospinning 

**Figure 1:**
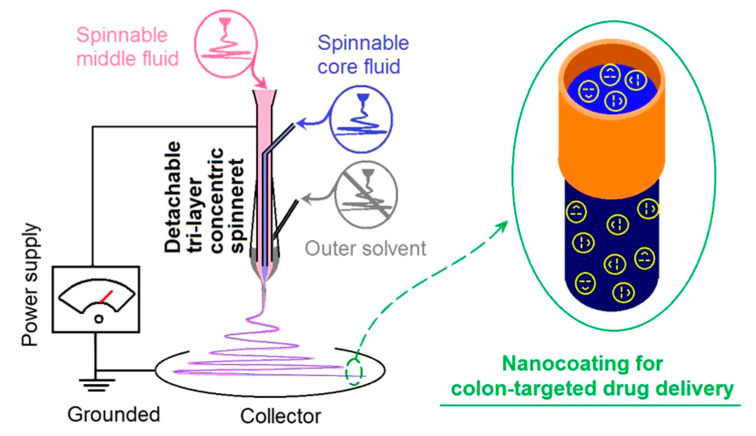
A diagram of the modified coaxial electrospinning.

A schematic of the homemade triaxial electrospinning system is given in Figure 1. As all other types of electrohydrodynamic atomization (EHDA) processes such as coaxial electrospinning and electrospraying, triaxial electrospinning has the similar essential four parts, i.e., fluid-driven pump, high-voltage generator, collector, and spinneret [51,52]. What makes differences are the spinneret and the corresponding numbers of syringe pumps. For a traditional single-fluid blended electrospinning, a syringe pump is exploited to quantitatively transfer the only spinnable working fluid to the monoaxial spinneret, often comprising a metal capillary [53]. For the coaxial processes, regardless of electrospinning or electrospraying, there are two pumps to simultaneously pump the core and shell working fluids to the double-layer concentric spinneret/spraying head [54,55,56,57,58]. Thus, it is expected that three pumps must be the prerequisite for quantitatively driving the three different working fluids to the core, middle, and outer layer inlets of the spinneret at the same time. Certainly, other apparatuses can be added or combined with the electrospinning systems to expand their capability of creating novel nanomaterials [59,60,61,62,63,64,65,66,67]. 

When the three-layer working fluids are all electrospinnable, the tri-fluid process is a typical triaxial electrospinning, and the products should be tri-layer core–shell fibers [68]. However, when the outer layer fluid is a pure solvent for lubricating the working process for a high-quality product, the nano products are double-layer core–shell fibers, and the tri-fluid process is a modified triaxial electrospinning [43,44,45,46,47,48,49,50]. The essential meaning is that the outer solvent provides air–solvent and solvent–polymer solution interfaces, instead of a previous air–polymer solution interface, and thus the “dynamic atomization” process is completely modified. The modified triaxial electrospinning has its own merits in creating core–shell nanofibers and implementing a surface coating at the nanoscale. 

**Figure 2:**
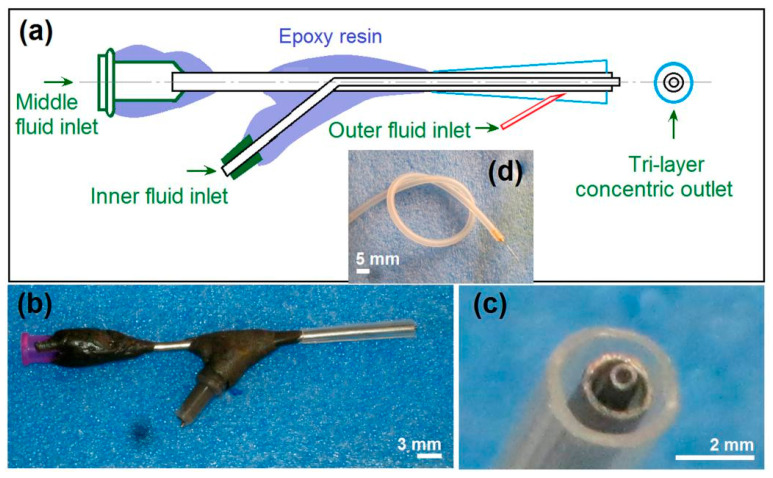
The detachable tri-layer concentric spinneret: (**a**) The design details of the spinneret with three inlets for the three working fluids and a common tri-layer concentric outlet; (**b**) a digital picture of the full view of the spinneret; (**c**) a digital image of the tri-layer concentric outlet. (**d**) A sharp needle with a highly elastic silica tubing for transferring the outer solvent, which has an outer diameter and a wall thickness of 0.3 and 0.05 mm, respectively.

The spinneret is the most important component in an electrospinning system, and often the spinneret’s inner structure determines the name of electrospinning processes, for example, a concentric spinneret means a coaxial process, and an eccentric spinneret represents side-by-side electrospinning [69]. In this study, a detachable triaxial spinneret was developed to lead the three fluids to the spinneret along a previous publication [70]. The design details of the spinneret with three inlets for the three working fluids and a common tri-layer concentric outlet are exhibited in Figure 2a, and digital pictures of the full view of the spinneret and the tri-layer concentric outlet are shown in Figure 2b,c, respectively. The double-layer metal spinneret comprised one metal capillary (having an outer and a wall thickness of 0.62 and 0.15, respectively) nested into another metal capillary (having an outer and a wall thickness of 1.84 and 0.25, respectively). A tapering polypropylene (PP) tube (its inner diameter varied from 2.50 to 1.84 mm with a fixed thickness of 0.3 mm) comprised the outer layer of the tri-layer concentric spinneret (Figure 2c). A sharp needle with a highly elastic silica tubing for transferring the outer solvent has an outer diameter and a wall thickness of 0.3 and 0.05 mm, respectively (Figure 2d). The outer layer solvent was guided into the spinneret simply through inserting the metal needle through the PP tubing.

**Figure 3:**
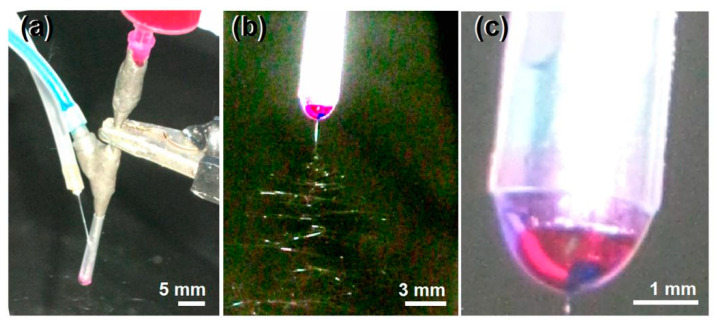
Observations about the modified triaxial electrospinning: (**a**) A digital picture of the connections of three working fluids and the high voltage with the spinneret; (**b**) a digital image showing a typical preparation process of the CSFs; (**c**) a typical compound tri-layer Taylor cone.

Shown in Figure 3a is a digital image of the connections of three working fluids and the high voltage with the spinneret. The plastic syringe holding the middle fluid was directly inserted into the tri-layer concentric spinneret. The core drug-ES100 co-dissolving solution was guided to the spinneret through an elastic silicon tubing. The outer layer solvent was led to the spinneret through a sharp needle, which could penetrate into the PP tapering tube. The high-voltage electrical energy was converted to the working fluid through an alligator clip. Under the optimized conditions, a typical working process for creating the CSFs is shown in Figure 3b. The classic three steps of electrospinning can be clearly discerned, i.e., the Taylor cone, the straight fluid jet, and the whipping and bending loops with a gradual increase in diameter due to electric repelling. The compound Taylor cone is exhibited in Figure 3c, in which the blue core layer was encapsulated by the red middle layer, and, in turn, encapsulated by the outer solvent mixture layer. A strange phenomenon is the color of the outer layer solvent mixture, which should be slightly blue. However, the diffusion of the red basic fuchsin molecules from the middle ES100 solution made a very light purple color. 

### 3.2. The Morphologies and Inner Structures of the MCFs and CSFs 

**Figure 4:**
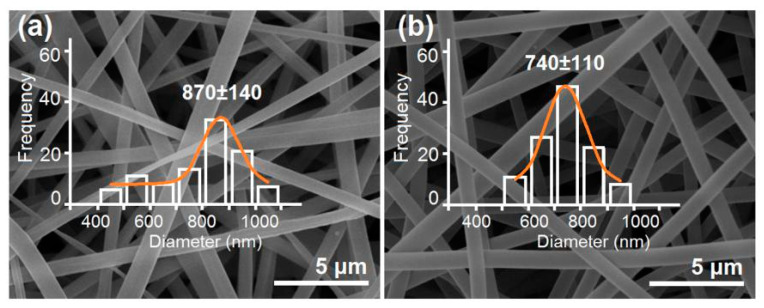
The SEM images of MCFs (**a**) and the CSFs created using the modified triaxial electrospraying (**b**).

The SEM features of the MCFs and the CSFs created using the modified triaxial electrospraying are shown in Figure 4a,b, respectively. It is clear that all the nanofibers have a smooth surface and a fine linear morphology free of beads-on-a-string or spindles-on-a-string phenomena. The reasons should be attributed to the fine electrospinnability of ES100 solutions under the selected working conditions. The MCFs had an average diameter of 870 ± 140 nm by estimation, and, by contrast, the CSFs had an average diameter of 740 ± 110 nm. Although the total fluid flow rates of the modified tri-axial electrospinning are larger than the fluid flow rate of the monoaxial single-fluid process, the CSFs had a smaller diameter than the MCFs. The reasons should be attributed to the following two reasons. One is that the outer solvent mixture was able to effectively retard the formation of the semi-solid substance around the fluids’ jets, and thus keep them from being drawn under the electrical field for a longer time period [63]. The other is that the applied voltage for creating the CSFs was slightly higher than that for producing the MCFs. 

**Figure 5:**
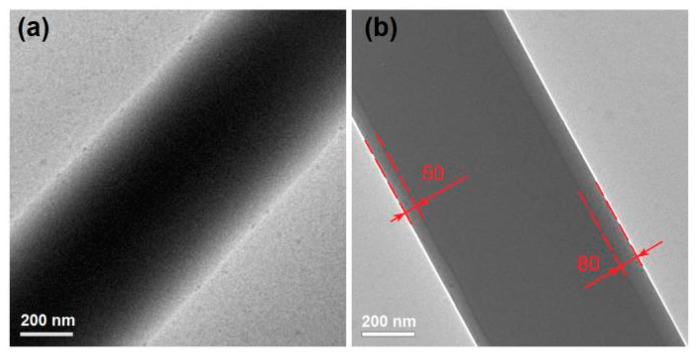
The TEM images of MCFs (**a**) and the CSFs created using the modified triaxial electrospraying (**b**).

The TEM images of the prepared MCFs using single-fluid blended electrospraying and the CSFs created using the modified triaxial electrospraying are shown in Figure 5a,b, respectively. Just as anticipated, the MCF nanofiber has a monolithic composite feature with the aspirin molecules highly homogeneously distributed all over the ES100 matrices. This can be reflected by the gradual change in gray level from the fiber’s center to the boundary, which is a direct result of the thicknesses of different places at the nanofiber. The CSF nanofiber has an obvious core–shell nanostructure, which has an average thickness of (80 + 50)/2 = 65 nm. The core section has an estimated diameter of 580 nm.

### 3.3. The Physical State of Aspirin and Its Compatibility with ES100

**Figure 6:**
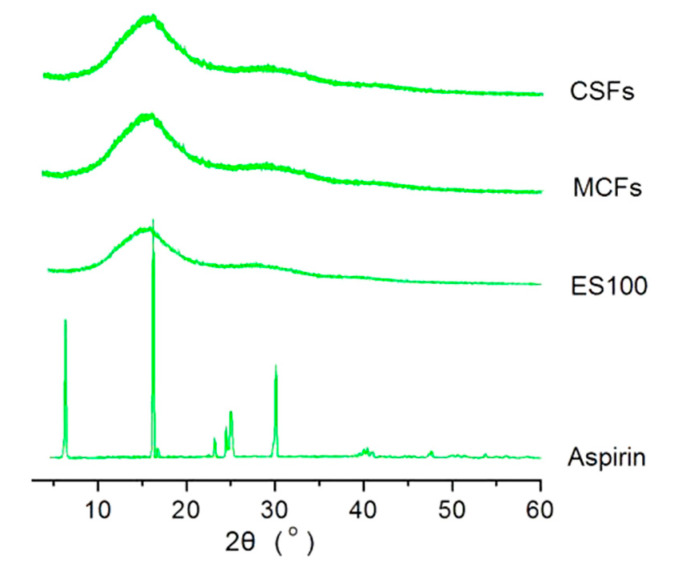
The XRD patterns of the raw materials (aspirin and Eudragit S100 (ES100)) and also their nanofibers MCFs and CSFs from different electrospinning processes.

The XRD patterns of the crude materials ES100, aspirin, and their nanofibers MCFs and CSFs are included in Figure 6. The curve of aspirin indicates that the crude white aspirin powders are typically crystalline due to the sharp peaks in its patterns. These sharp peaks are Bragg responses that reflect X-ray beams at certain angles of incidence (θ), verifying the presence of lattice planes and in turn the polycrystalline property of aspirin powders. The filament-forming matrix ES100 has no sharp peaks except two blunt halos, giving a hint that it is an amorphous material. The nanofibers MCFs and CSFs from the two different electrospinning processes have a similar XRD curve, just as the pattern of ES100. These phenomena suggest that all the aspirin molecules are converted into an amorphous state to co-exist with the ES100 molecules in the two types of nanofibers.

**Figure 7:**
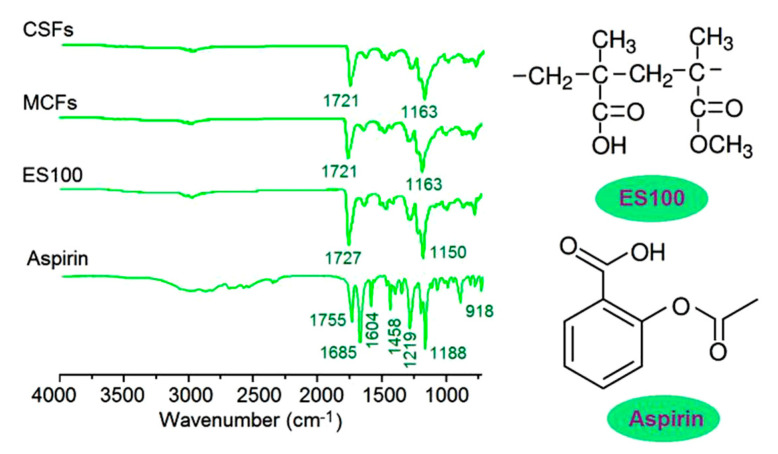
The ATR-FTIR spectra of the raw materials (aspirin and ES100) and also their nanofibers MCFs and CSFs from different electrospinning processes.

Attenuated total reflection (ATR)-FTIR spectra of the starting materials aspirin, ES100, and their nanofibers MCFs and CSFs are shown in Figure 7. The most important characteristic peaks of aspirin are at 1755, 1685, and 1604 cm⁻^1^, which are responses from the -C=O groups and benzene rings [71]. As for the filament-forming matrix and also the drug carrier ES100, the main characteristic peaks are at 1727 and 1150 cm⁻^1^, which are responses from the –C=O groups and –C–O–C groups. In their electrospun nanofibers MCFs and CSFs, many peaks of aspirin are almost disappeared, which indicate that ES100 and aspirin are compatible. This can be deduced from their molecular formula. In an aspirin molecule, there are two –C=O groups and a benzene ring. The –C=O groups can form hydrogen bonds with the –OH groups in the ES100 molecules, and vice visa, the –OH groups in the aspirin molecules can form hydrogen bonds with the –C=O groups in the ES100 molecules. 

### 3.4. The Functional Performances of Aspirin Colon-Targeted Extended-Release Profiles 

**Figure 8:**
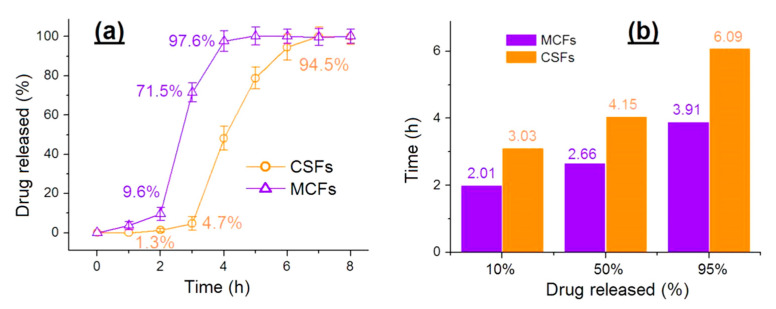
The in vitro drug-release performances: (**a**) Accumulative aspirin release as a function of drug-release time points; and (**b**) the time needed for achieving a certain percentage of aspirin loaded in the prepared nanofibers.

The aspirin-release profiles from the two sorts of nanofibers MCFs and CSFs are shown in Figure 8. The performances of aspirin release can be compared mainly from two aspects: The delayed-release time period for a colon-targeted effect and a prolonged release for an extended-release effect. In the first two hours in the acid dissolution bulk solutions, MCFs and CSFs released 9.6 ± 3.4% and 1.3 ± 0.8% of the loaded drug (Figure 8a), suggesting that the CSFs had the better performances in terms of preventing aspirin release in the stomach, which is reported to directly damage the mucosal surface of the stomach [72]. At the third hour, MCFs and CSFs released 71.5 ± 4.8% and 4.7 ± 3.2% of the loaded aspirin. The pulsatile release of MCFs may lead to another side effect due to overdose, i.e., cytotoxicity in the circulation system. In addition, after 4 h, MCFs released 97.6 ± 5.2% of the loaded aspirin, whereas CSFs released 94.5 ± 6.4% of their aspirin after 6 h, suggesting a longer time period and a more sustained manner to extend the aspirin release in the neutral conditions. 

From another angle, the advantages of CSFs over MCFs can be seen in Figure 8b. A percentage of 10% release costs MCFs and CSFs 2.01 and 3.03 h, respectively. CSFs have the better delayed-release effect after possible oral administration. A percentage of 50% and 95% costs MCFs 2.66 and 3.91 h, respectively. By comparison, the time periods for CSFs are 4.15 and 6.09 h, considerably better than those for MCFs in terms of sustained-release time periods.

To disclose the influences of the ES100 shell layer on the drug-release kinetics, the release data of MCFs and CSFs were regressed using the Peppas Equation [73], as shown below.

**Figure 9:**
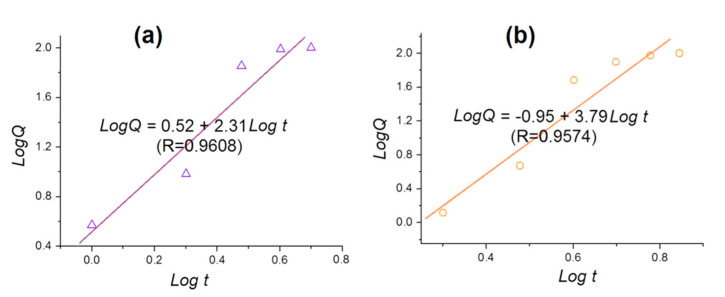
The regressed equations of aspirin release data of nanofibers from different electrospinning processes according to the Peppas equation: (**a**) MCFs; (**b**) CSFs.

*Q* = *kt*^n^

where *Q* represents the accumulative release percentage of aspirin, *t* represents the release time, *k* is a constant, and the power n is an important parameter to indicate the drug release mechanism. Often, when the n value is smaller than 0.45, it is suggested that the drug molecules are released by a typical Fickian mechanism. By contrast, when the n value is larger than 0.9, the drug is regarded to be released through an erosion mechanism. A value of n between 0.45 and 0.90 indicates a combined mechanism of diffusion and erosion. The regressed equation for MCFs is *LogQ = 0.52 + 2.31 Logt* (*R* = 0.9608) (Figure 9a). A bigger n value of 2.31 > 0.90 suggests that aspirin released from the MCFs through an erosion mechanism, i.e., the release of aspirin is controlled by the dissolution or erosion rates of ES100. The regressed equation for CSFs is *LogQ* = −0.95 + 3.79*Logt* (*R* = 0.9574). Similarly, a bigger n value of 3.79 > 0.90 suggests that aspirin released from the CSFs also through an erosion mechanism. This suggests that the shell ES100 layer has no influence on the drug release mechanism.

## 4. Conclusions

Based on the pH-sensitive polymer ES100, a modified triaxial electrospinning was developed for preparing polymeric core–shell structural nanofibers (CSFs), in which aspirin was successfully encapsulated. Traditional types of monolithic composite nanofibers (MCFs) were fabricated using a single-fluid blending process for comparison. The MCFs and CSFs have a similar linear morphology, a smooth surface, and an estimated average diameter of 870 ± 110 and 740 ± 110 nm, respectively. The CSFs had a clear core–shell structure with a shell thickness of 65 nm. Aspirin has fine compatibility with ES100, as disclosed by XRD patterns and ATR-FTIR results. However, the CSFs show considerable advantages over the MCFs in providing the desired drug-controlled-release profiles, although both of them freed the drug in an erosion mechanism. The former furnished a longer time period of time-delayed release (3.03 vs. 2.01 h) and a smaller portion release (9.6 ± 3.4% vs. 1.3 ± 0.8%) during the first two-hour acid condition for protecting the stomach membranes, and also showed a longer time period of aspirin-extended-release for avoiding overdose-caused cytotoxicity in the circulation system (6.09 vs. 3.91 h). The present protocols provide a polymer-based process-nanostructure-performance relationship to optimize the reasonable delivery of aspirin.

## Figures and Tables

**Table 1:** Parameters of the monolithic composite nanofibers (MCFs) and core–shell structural nanofiber (CSFs).

No.^a^	Electro-Spinning	Voltage (kV)	Flow Rate of Fluid (mL/h)			Morphology/ Structure	Drug Loading (wt. %)
			Outer	Middle	Inner		
MCFs	Single-fluid	13	2.0			Linear/Monolithic	25.0%
CSFs	Triaxial	15	0.5	0.6	1.4	Linear/Core–shell	19.4%

^a^ MCFs and CSFs represent monolithic composite nanofibers and core–shell structural nanofibers, respectively.
